# Maternal Emotional Availability Supports Child Communicative Development Regardless of Child Temperament—Findings From the FinnBrain Birth Cohort Study

**DOI:** 10.1111/infa.12649

**Published:** 2025-01-24

**Authors:** Denise Ollas‐Skogster, Riikka Korja, Akie Yada, Elina Mainela‐Arnold, Hasse Karlsson, David J. Bridgett, Pirkko Rautakoski, Linnea Karlsson, Saara Nolvi

**Affiliations:** ^1^ Department of Speech and Language Pathology Åbo Akademi University Turku Finland; ^2^ FinnBrain Birth Cohort Study, Turku Brain and Mind Center, Department of Clinical Medicine University of Turku Turku Finland; ^3^ Department of Psychology and Speech‐Language Pathology University of Turku Turku Finland; ^4^ Centre of Excellence in Learning Dynamics and Intervention Research (InterLearn) University of Jyväskylä Jyväskylä Finland; ^5^ University of Turku Turku Finland; ^6^ Department of Psychology Faculty of Education and Psychology University of Jyväskylä Jyväskylä Finland; ^7^ Department of Education Faculty of Education and Psychology University of Jyväskylä Jyväskylä Finland; ^8^ Department of Psychiatry University of Turku and Turku University Hospital Turku Finland; ^9^ Centre for Population Health Research University of Turku and Turku University Hospital Turku Finland; ^10^ Department of Psychology University of Nevada Reno Nevada USA; ^11^ Unit of Public Health Department of Clinical Medicine University of Turku Turku Finland; ^12^ Department of Child Psychiatry Turku University Hospital Turku Finland

**Keywords:** communicative development, emotional availability, infant, temperament, toddler, vocabulary

## Abstract

The interplay of emotional availability (EA) and child temperament in association with early language development is understudied. We explored associations between maternal EA and infant communicative development and possible moderations by child temperament. Participants were 151 mother‐child dyads from the FinnBrain Birth Cohort Study. Path models of associations between 8‐month maternal EA and 14‐month communicative development and moderation by infant temperament traits were created using SEM. Results show that EA positively predicted a latent variable of communicative development at 14 months. No direct longitudinal effect of EA on 30‐month vocabulary was found. Child surgency/extraversion at 6 and 12 months significantly predicted 14‐month communicative skills. Temperament did not moderate the association between EA and communicative development. Findings underscore the additive role of maternal caregiving and early surgency/extraversion in predicting early communicative development. The emotional aspects of parenting should be acknowledged as contributors to early communicative development in future studies.

## 1 Introduction

Infant communicative development takes place in interaction with others, and therefore the quality of early interactions experienced by the infant is relevant to investigate in the context of communicative development (Tamis‐LeMonda, Bornstein, and Baumwell 2001). Both infant characteristics and factors related to the interaction partner are likely to contribute to the quality of the interaction. Consequently, it is important to study the contributions from both members of the dyad to infant communicative development and how these factors possibly interact. This is notable because early communication and language development predict later language skills, and have implications for other later developmental outcomes such as socio‐emotional functioning and academic achievement (Hohm et al. 2007). Furthermore, the concept of emotional availability—referring to caregiving quality in terms of emotional connection in the mother‐child dyad—has frequently been overlooked in the context of communicative development in past studies in favor of studying caregiving from a narrower viewpoint.

### 1.1 Caregiving Quality and Communicative Development

Several studies report that parent caregiving quality predicts communicative development (Leigh, Nievar, and Nathans 2011; Nozadi et al. 2013; Tamis‐LeMonda, Bornstein, and Baumwell 2001). Similar findings have been reported in a Finnish sample, suggesting that across cultures, caregiving quality is an important contributor to early communicative development (Paavola et al. 2006). Nevertheless, the terms used for caregiving quality and what is included in the definitions vary between studies. Most typically, maternal responsiveness or sensitivity are the foci of interest in studies of caregiving quality. Sensitivity generally refers to the parent's awareness of the child's signals and needs (Ainsworth et al. 2014), and responsiveness is often thought of as the behavioral consequence of sensitivity—reacting to meet the needs of the child (Biringen and Easterbrooks 2012). Findings across studies consistently report sensitivity/responsiveness to be positively related to language development regardless of socioeconomic status (SES) or ethnicity of the sample (Pungello et al. 2009). For example, Leigh, Nievar, and Nathans (2011) found that higher maternal sensitivity during the first and second year of life predicted better expressive language skills at two and 3 years of age. They also found that concurrent relations between sensitivity and child language were stronger as the child grew older, with the strongest relationship at the last assessment point at 3 years (Leigh, Nievar, and Nathans 2011). Tamis‐LeMonda, Bornstein, and Baumwell (2001) reported positive associations between maternal responsiveness at 9 and 13 months postpartum and the achievement of infant expressive language milestones. Associations between maternal sensitivity and both expressive and receptive language skills in preschool‐aged children have also been found (Keown, Woodward, and Field 2001). Additionally, maternal responsiveness demonstrated positive associations with non‐verbal communicative behaviors such as symbolic play (Bornstein and Tamis‐LeMonda 1997). Although findings have been fairly consistent across studies, there are reports where responsiveness did not significantly associate with gesturing or language outcomes in infancy and toddlerhood (e.g., Bruce et al. 2022).

Another caregiving‐related concept studied in relation to communicative development is scaffolding (Dieterich et al. 2006; Landry et al. 1997). Scaffolding generally refers to providing support adapted to the individual child's needs to help them accomplish goals they would not be able to accomplish on their own, while allowing and encouraging independent problem‐solving (Wood, Bruner, and Ross 1976). A subcategory is verbal scaffolding, referring to rich verbal input from the parent encouraging independent problem solving by the child (Dieterich et al. 2006). Particularly, verbal scaffolding is regarded as supportive of communicative development (Dieterich et al. 2006; Landry et al. 2002). Scaffolding has some overlap with the responsiveness concept, and some consider it a subcategory of it (Landry, Smith, and Swank 2006; Mermelshtine 2017; Tamis‐LeMonda, Kuchirko, and Song 2014).

Further, the concept of structuring, used in our study, has commonalities with scaffolding, but where scaffolding describes general parenting, structuring refers to the parent's interactive, behavioral structuring of their and the child's actions in the immediate situation at hand (Saunders et al. 2015). It is not merely assessing the parent's ability to support child development, but the parent's interactive ability to structure the situation according to the child's signals. Optimal structuring helps the child be active and autonomous, and understand what is expected of them in a situation (Biringen 2000).

Whereas positive aspects of caregiving have been considered in prior studies, the negative aspects of caregiving have been studied less commonly in relation to language development. One aspect of negative caregiving is parental intrusiveness. Parental intrusiveness typically refers to caregiver over‐involvement, and the inability to let the child take the lead or react adequately to the child's signals (Laake and Bridgett 2018; Saunders et al. 2015). Some studies have found negative associations between higher levels of parent intrusiveness and child language (Bruce et al. 2022; Keown, Woodward, and Field 2001; Pungello et al. 2009). However, others have found the effect to disappear when simultaneously accounting for positive caregiving (Tamis‐LeMonda et al. 2004) or the associations to appear only at specific age points (Cabrera, Shannon, and Tamis‐LeMonda 2007).

In this study, we measured caregiving quality during mother‐child interaction using the emotional availability (EA) construct (Biringen 2000; Biringen and Easterbrooks 2012; Saunders et al. 2015). Within the EA scales framework, EA is defined as the dyad's ability to form a genuine positive emotional connection and adapt to and regulate negative emotions (Biringen and Easterbrooks 2012). High EA characterizes a healthy, positive, and mutually enjoyable interaction and relationship. The EA framework includes the aspects of *sensitivity*, but also *structuring, non‐intrusiveness,* and *non‐hostility* (Biringen 2000). These aspects are consistently related to child emotion regulation (Frick et al. 2018) and empathy development (Moreno, Klute, and Robinson 2008), as well as mental health and behavior problems (Easterbrooks, Bureau, and Lyons‐Ruth 2012); factors that are also commonly linked to communicative development (Moreno, Klute, and Robinson 2008; Reilly and Downer 2019). Although measures of sensitivity/responsiveness, scaffolding, or structuring, and intrusiveness, corresponding in varying degrees to the equivalent subscales involved in EA, have been explored in relation to communication development, we are not aware of previous studies that have used the concept of EA and the combination of the subscales into a latent variable depicting overall caregiving quality. Thus, using the EA framework enables exploration of the effects of a broader, multifaceted conceptualization of caregiving including both emotional and behavioral dimensions.

### 1.2 Temperament and Communicative Development

Whereas caregiving quality is an important parental factor influencing early communicative development, temperament is one infant‐related factor that plays a role in shaping child language. Temperament is commonly defined as individual differences in emotional, motor, and attentional reactivity and self‐regulation (Rothbart and Bates 1998). The temperament framework of Rothbart contains three overarching factors: surgency/extraversion, negative emotionality, and self‐regulation (Gartstein and Rothbart 2003). Surgency/extraversion is a temperament trait generally involving characteristics such as pleasure related to high‐intensity stimuli, high approach, impulsivity, and activity level (Putnam, Ellis, and Rothbart 2001). Negative emotionality, in turn, involves characteristics related to expression of negative emotions such as sadness, frustration, and fear. Self‐regulation is defined as the processes that control reactivity. That is, self‐regulation involves controlling attention, approach, and inhibition. Early manifestations of self‐regulation involve attentional processes that support the emergence of effortful self‐regulation of behavior and emotion (i.e., effortful control; Bridgett et al. 2011; Putnam, Ellis, and Rothbart 2001). Self‐regulation (Bruce et al. 2022; Dixon and Smith 2000) and surgency/extraversion (Bruce et al. 2022; Dixon and Smith 2000; Laake and Bridgett 2014) have previously been reported to be positively associated with communicative development. In turn, there is more variability in the findings regarding negative emotionality. Some studies have reported negative associations between infant language development and high negative emotionality (Bloom and Capatides 1987; Kubicek and Emde 2012; Salley and Dixon 2007), whereas others have reported opposite relations (Molfese et al. 2010; Moreno and Robinson 2005; Robinson and Acevedo 2001) or no association at all (Ollas‐Skogster et al. 2023).

The associations between temperament and communicative skills are also hypothesized to, at least to some extent, be due to the influence of temperament traits on the child's interaction with others (Laake and Bridgett 2014). For instance, it is thought that child temperament may influence the adults' interaction style with the infant (Conture, Kelly, and Walden 2013). It may be that adults interact more with infants who show high surgency/extraversion, resulting in larger amounts of communicative stimulation supporting the learning of communicative skills (Laake and Bridgett 2014). Furthermore, infant self‐regulation, including higher attentiveness and duration of orienting, is suggested to support communicative development through the improved ability to focus on interactions with the caregiver and acquire communicative skills by observing and engaging in interaction with others (Gartstein, Crawford, and Robertson 2008). The infant's better ability to focus attention possibly also serves as a motivator for the partner to continue the verbal interaction (Gartstein, Crawford, and Robertson 2008).

The positive associations between negative emotionality and language development also have been explained by displays of negative emotions resulting in the engagement of the caregiver, and consequently more interactive and communicative stimulation (Moreno and Robinson 2005). This possibly shows that negative emotions can also be seen as a prelinguistic infant's regulatory strategy related to the interaction with the adult (Moreno and Robinson 2005). For this to be beneficial for communicative development, adult sensitivity to these signals is required, again underscoring the codependence in the interactional relationship within the dyad. However, more emotionally negative infants have also been reported to make fewer bids to initiate joint attention (Salley and Dixon 2007); that is, they may initiate communication with the caregiver less frequently.

### 1.3 Caregiving Quality, Temperament, and Communicative Development

To summarize, there is a growing body of literature reporting associations between temperament as well as caregiving quality and communicative development, and these findings are often explained by factors related to dyadic interaction. Given that parents and children act together to form a relationship (Collins et al. 2000), and the temperament of the infant can influence the qualities of the caregiving (Kochanska et al. 2004), and possibly also maternal language used in interaction with the infant (Vernon‐Feagans et al. 2008), surprisingly few studies have focused on the moderating role of temperament in explaining the association between caregiving quality and language development.

To describe the few exceptions, Karrass and Braungart‐Rieker (2003), Laake and Bridgett (2018), and Nozadi et al. (2013) found caregiving quality and temperament traits to interact in predicting language skills in toddlerhood (14–18 months of age). In contrast, Gartstein, Crawford, and Robertson (2008) and Bruce et al. (2022) found caregiving factors and temperament to have only independent effects on communicative skills in infancy and toddlerhood (6–24 months). Karrass and Braungart‐Rieker (2003) found maternal responsiveness to positively predict total language ability when child fearfulness was low. Maternal responsiveness also predicted stronger language abilities in boys with a low level of smiling and laughter—an aspect of surgency/extraversion. Karrass and Braungart‐Rieker (2003) suggest that infants with low emotionality of both negative and positive quality may be advantaged in engaging in communication with the caregiver, and thereby better equipped to utilize the input by highly responsive caregivers (Karrass and Braungart‐Rieker 2003). Conversely, Laake and Bridgett (2018) found that high surgency/extraversion at 10 months was predictive of better expressive language at 14 months only in the context of higher maternal support. Infants higher in positive as well as negative emotionality benefited from lower maternal intrusiveness, but unexpectedly, in infants lower in negative emotionality, higher maternal intrusiveness had positive effects on expressive language. The authors suggested that in a context where the infant's temperament does not favor eliciting interaction, even intrusive maternal initiatives can support communicative development as more intrusive parents may initiate more interaction with infants than parents lower in intrusiveness. Finally, Nozadi et al. (2013) found that maternal sensitivity influenced boys' expressive language development in toddlerhood indirectly through its role in regulating anger expression.

To conclude, existing studies have mostly underscored the role of infant affectivity as a moderator of the association between caregiving quality and communicative development. Furthermore, preverbal gesturing has seldom been involved in the assessment of infant communicative skills in this context, although it is a precursor of verbal language (Iverson and Goldin‐Meadow 2005) and offers an opportunity to study the earliest forms of emerging infant communication.

### 1.4 The Current Study

The aim of the current study was to investigate how early maternal caregiving quality assessed as EA at 8 months predicted preverbal communicative behaviors (at 14 months) and early verbal language skills (at 14 months and 30 months). An additional aim was to test whether infant temperament moderated the association between maternal EA and communicative skills. This study builds on the limited existing work in this area by using a wider perspective on caregiving quality involving all scales of EA including sensitivity, structuring, non‐intrusiveness, and non‐hostility. It also involves gesturing as a measure of early preverbal communicative skills and explores associations between overarching latent concepts of both caregiving quality and infant communicative skills. We used structural equation modeling (SEM) to create a path model of a latent EA‐variable and temperament traits predicting a latent variable of 14‐month communicative skills and observed 30‐month expressive vocabulary. The use of latent variables allows for more statistically sound assessments of the phenomena of caregiving and communicative development on a general level. Furthermore, the use of EA offers a new, emotional viewpoint on caregiving rarely utilized in this context before. Finally, we also tested possible moderation effects of temperament traits (surgency/extraversion, negative emotionality, fear, and emerging self‐regulation) on the relations between EA and communicative skills.

Based on prior research, we hypothesized that emotional availability at 8 months would positively predict children's communicative skills at 14 months and again at 30 months of age. We expected emotional availability and temperament traits to interact in predicting communicative skills in infancy and toddlerhood. More specifically, surgency/extraversion, negative emotionality, fear, and emerging self‐regulation were considered as possible moderators based on prior studies. We expected that higher emotionality of both positive and negative quality paired with high quality caregiving would be positively associated with communicative development. We assumed emotional expressions and bids would be particularly efficient in initiating interaction with an emotionally available caregiver. Similarly, we expected high emerging self‐regulatory ability in infants to be positively associated with communicative development accompanied by high‐quality caregiving, since this trait could support infant involvement in joint activities further enhanced by high‐quality caregiving. As previous findings on interactions between positive and negative emotionality and caregiving quality as predictors of communicative outcomes are mixed and corresponding findings regarding self‐regulation and caregiving quality are, to our knowledge, missing, these analyses were considered exploratory.

## 2 Method

### 2.1 Participants

The study involved 151 families participating in the FinnBrain Birth Cohort Study (Karlsson et al. 2018). Compared to corresponding studies in the field that have found statistically significant associations (Bruce et al. 2022; Gartstein, Crawford, and Robertson 2008; Karrass and Braungart‐Rieker 2003; Laake and Bridgett 2018; Nozadi et al. 2013), the current sample size is medium‐sized. It is a convenience sample derived from a large cohort study representative of the geographically defined target population (Karlsson et al. 2018) and based on the availability of relevant data. Since the data are part of a large longitudinal cohort with a variety of research questions and were already collected at the time of planning this study, we could not calculate a priori power analyses for this study specifically. Nevertheless, the sample size is considered sufficient for detecting medium‐sized and clinically relevant effects. The study follows the guidelines of the Declaration of Helsinki. Parents or guardians of each child provided written informed consent prior to the child participating in any study procedure. The cohort study protocol has received approval from the Ethics Committee of the Hospital District of Southwest Finland. All families who took part in laboratory visits for temperament and EA observations as well as provided at least one of the communicative development questionnaires at either 14 months or 30 months of age were included in the sample. The participating families were for the most part included in the FinnBrain “Focus cohort.” The families in the Focus cohort are followed using comprehensive multidisciplinary assessments during the first years of the child's life (Karlsson et al. 2018). Demographic characteristics regarding the families can be seen in Table 1. The demographic features of the current sample correspond quite well to the characteristics of the entire cohort (Karlsson et al. 2018), except for children being primiparous more often in the current sample. Regarding attrition, the subsample that had not filled in the 30‐month language development questionnaire did not differ significantly from the rest of the sample regarding any of the demographic characteristics. Five children were born preterm, one in gestational week 34 and the rest in week 36. The final models reported were run without the prematurely born infants for comparison and did not differ from the original versions. Hence, the prematurely born infants were involved in the final analyses. In this sample, monolingualism was defined as being exposed to another language besides the mother tongue < 20%. Bilingual families completed the CDI in Finnish. In bilingual families, the families most typically spoke Finnish and Swedish, both national languages of Finland. No significant differences between bi‐ and monolingual infants in communicative skills were found in independent samples *t‐*tests, and consequently, all infants were included in further analyses.

**TABLE 1:** Demographic characteristics of the sample.

	Mean (*SD*)	%	Range
Child characteristics			
Gender, girl		51.0	
First‐born		63.6	
One sibling		27.2	
≥ 2 siblings		9.3	
Gestational age at delivery	39.8 (1.34)		34.4–42.3
Age at 8‐month laboratory visit (months)	8.2 (0.4)		7.0–9.6
Family characteristics			
Family language status			
Monolingual family		64.9	
Bilingual family		5.3	
Data missing regarding bilingualism		29.8	
Maternal educational level			
University degree		39.7	
Polytechnic/applied university degree		38.4	
Vocational training/high school/basic education		19.8	
Data missing regarding maternal education		2.0	
Maternal age at delivery	31.2 (4.0)		21–44
Paternal age at delivery	32.2 (4.6)		21–45

### 2.2 Procedures

Families were recruited in the maternal welfare clinics at gestational week 12 through personal contact with a research nurse. Background information regarding the families was collected using questionnaires at gestational week 14 and supplemented with information from later data points when needed. Additionally, information regarding child gender and gestational age at birth from the maternity ward was retrieved from the wellbeing services county of Southwest Finland (VARHA) records. When the child was 8 months of age, the families in the focus cohort were invited to a laboratory visit at the FinnBrain research site in Turku, where experimental assessments of temperament, as well as emotional availability in the mother‐child dyad, were carried out by trained psychologists or advanced‐level psychology master's students. Maternal reports on infant temperament were collected at 6 and 12 months of age, and questionnaires regarding child communicative development were filled out by families when the child was 14 months and 30 months old.

### 2.3 Measures

#### 2.3.1 Communicative Development

Communicative skills were assessed using the Finnish versions of the Mac‐Arthur‐Bates Communication Development Inventories (CDI), the Words and Gestures‐version at 14 months, and the Words and Sentences‐version at 30 months (Fenson et al. 1994; Lyytinen 1999). The CDI is a commonly used, reliable, and valid parent report of infant and toddler communicative development. In two cases, the Swedish version (Eriksson and Berglund 1999) was used in monolingual Swedish‐speaking Finnish families. Since the sample included two Swedish CDI‐versions in addition to the Finnish ones and the total sum scores differ between the versions, we used the percent scores (percent of maximum score) for the language variables to be able to use both versions in the same analyses. We used the sum scores for words understood (receptive vocabulary) and spoken (expressive vocabulary), as well as the use of gestures at 14 months, and the sum score for total vocabulary (expressive) at 30 months. The actions and gestures assessment consists of lists of the first communicative gestures, games and routines, actions with objects, pretending to be a parent, and imitating other adult actions. The communicative gestures section includes options to check whether the gesture is used often, sometimes, or not at all by the infant, whereas the use of the remaining actions and gestures listed is assessed on a yes/no basis. The sum score for the total use of gestures was used (McDonald's *ω* = 0.79). Vocabulary sum scores are attained from a checklist of words in different semantic categories, where the parent checks whether the infant understands (*ω* = 0.96) or understands and produces (*ω* = 0.89) the word in the Words and Gestures version, whereas no distinct assessment of receptive vocabulary, only production of the words, is included in the Words and Sentences‐version (*ω* = 0.99). The final SEM‐models reported were run with and without possible outliers (one observation for the gestures measure and one for expressive vocabulary at 14 months that had *z*‐scores of ± 3 SD). Models did not change significantly due to excluding outliers. The only observed difference was the significance value of a negative association between surgency/extraversion at 6 months and EA in the final model decreasing below the significance threshold. Hence, outliers were kept in further analyses.

#### 2.3.2 Emotional Availability

Emotional availability was assessed using the fourth edition of the Emotional Availability Scales (EAS, Biringen 2008). The assessment was based on a 20‐min free play session between the infant and mother in a laboratory setting. The dyad was instructed to sit on the floor and play as they normally would at home. A set of age‐appropriate toys was available, and the dyad was instructed to use them if they wished. The session was videotaped and coded on six dimensions of emotional availability: four for the adult and two for the child. These dimensions are adult sensitivity, structuring, non‐intrusiveness, and non‐hostility, and child responsiveness and involvement (Saunders et al. 2015). In this study, we only used the adult scales from which a latent variable of total emotional availability was created. Sensitivity refers to how the mother strives to create a healthy, positive emotional connection with the child using different actions and emotions. Structuring is the adult's ability to scaffold the child by adapting the situation according to the child's abilities, creating opportunities for learning, and providing help when needed without restricting the child's autonomy. Non‐intrusiveness is the ability to let the child take the lead in the situation, permitting age‐appropriate independence, and not interfering or interrupting unless needed or requested by the child. Non‐hostility is the functional regulation of adult negative emotions and the absence of overt or covert displays of hostile emotions or behaviors, such as frustration, boredom, or aggression.

Dimensions are scored on a scale ranging from 1 to 7, where higher scores indicate warm and healthy emotional availability, whereas low scores are indicative of reasons for concern regarding the relationship (Saunders et al. 2015). Reliability was assessed in 10% of the cases. The intraclass correlation coefficients were as follows: sensitivity 0.80, structuring 0.72, non‐intrusiveness 0.85, and non‐hostility 0.70. In cases of differing assessments between coders, the coders negotiated and obtained consensus ratings for subsequent analyses.

#### 2.3.3 Temperament

##### 2.3.3.1 Observed Temperament

Episodes from the Laboratory Temperament Assessment Battery—Prelocomotor version (Lab‐TAB, Goldsmith and Rothbart 1999) were used to assess temperament at the infant age of 8 months. The Lab‐TAB is a standardized temperament assessment battery in which child reactions to different stimuli are videotaped and used as indicators of temperament dimensions (Planalp et al. 2017). In addition to child reactions, caregiver behavior/interference was coded for every temperament episode (0 = very inefficient parent behavior, does not follow instructions; 1 = parent mildly deviates from instructions; and 2 = parent behaves as instructed). Caregiver behavior was consequently controlled for in analyses involving the respective temperament episode. Coding of the videotapes was performed by trained coders.

The temperament dimension of Fear was evaluated during the Masks episode from Lab‐TAB. During the episode, the infant is presented with four different masks in order of increasing probability of eliciting a fear reaction. The episode is coded for four fear responses for each individual mask presentation: escape behaviors (0–3), facial fear (0–3), bodily fear (0–3), and fearful vocalizations (0–5) (intercorrelations ranging from 0.45 to 0.78, McDonald's *ω* = 0.90). The sum score, composed of the standardized scores for all responses, was used. The episode presented good inter‐rater reliability (Cohen's Kappa ranging from 0.73 to 0.83, with inter‐rater correlations of *r* = 0.95–0.98).

Task orientation/attentiveness, an aspect of emerging self‐regulatory capacity, was observed during the Blocks episode. During the episode, the infant is given a set of blocks to play with during a 3‐min session. The caregiver is instructed to be as uninvolved in the play as possible. During the episode, infant intensity of facial interest (0–3), duration of looking (0–2), and manipulation of stimuli (0–3), are coded for. A standardized sum score is calculated from the indicators (intercorrelation = 0.30, *ω* = 0.77). The manipulation indicator was excluded from the sum score in this case due to low variability within this indicator. The episode had adequate inter‐rater reliability (Cohen's *K* = 0.72–0.79, with inter‐rater correlations ranging from *r* = 0.47 to 0.99).

Evaluations of negative emotionality and self‐regulation were obtained from the Restraint episode, where the infant is given an interesting toy with which to play. Subsequently, the caregiver is instructed to start holding the infant's arms gently by their sides, which results in infant frustration because they can no longer reach the desired toy. This is continued for two episodes of 30 s with a break in between when the child can play with the toy again. We used the negative emotionality‐indicators: facial anger (0–3), facial sadness (0–3), and distress vocalizations (0–5) for the sum score (intercorrelation ranging from 0.48 to 0.84, *ω* = 0.89). Inter‐rater reliability was adequate (Cohen's *K* = 0.62–0.79, with inter‐rater correlations ranging from *r* = 0.64–99). Although not part of the original Lab‐TAB setup, we also used the same Restraint episode for coding self‐regulation by gaze aversion (Ollas‐Skogster et al. 2023). Gaze aversion is the earliest emerging indicator of self‐regulation in infancy (Rothbart et al. 2011). It was coded as the number of seconds spent looking away from the toy during the episode, and the variable was a sum of seconds looking away during all epochs. The reliability was at a good level (inter‐rater correlations ranging from *r* = 0.78–0.95).

##### 2.3.3.2 Reported Temperament

Parent reports of temperament were carried out at the infant ages of 6 and 12 months using the Infant Behavior Questionnaire – Revised (IBQ‐R, Gartstein and Rothbart 2003), which is a questionnaire on infant behavior in a variety of everyday situations. Mothers rated on a 7‐point scale from “never” to “always” how often they had observed a certain behavior in the child during the past week. From the questionnaires, 14 subscales and three broader factor dimensions were obtained: surgency/extraversion (McDonald's *ω* = 0.87–0.89 for 6 and 12‐month assessments, consisting of the subscales approach, vocal reactivity, high‐intensity pleasure, smiling and laughter, activity level, and perceptual sensitivity), negative emotionality (*ω* = 0.80–0.89, consisting of sadness, distress to limitations, fear, and falling reactivity) and emerging self‐regulation (*ω* = 0.85–0.87, consisting of low intensity pleasure, cuddliness/affiliation, duration of orienting, and soothability) are obtained (Gartstein and Rothbart 2003). In the current study, we used the three broad factors, as well as the subscale of Fear (*ω* = 0.83–0.84) to match the observed temperament measures used. Furthermore, the theoretical rationale for examining fear separately stems from previous literature suggesting that, for some individuals, the trajectory of fear reactivity might differ from other aspects of negative emotionality (e.g., Gartstein et al. 2010; Rothbart 2011).

#### 2.3.4 Covariates

To control for other variables that have been observed to relate to communicative development, we chose to include infant gender, maternal education level, number of siblings, and gestational age as covariates in the analyses (Berglund, Eriksson, and Westerlund 2005; Hoff‐Ginsberg 1998; Zerbeto, Cortelo, and Filho 2015).

Data regarding gender and gestational age were obtained from the Wellbeing Services County of Southwest Finland (VARHA) records. Mothers self‐reported their educational level in a questionnaire package during the first trimester of pregnancy. Educational level was coded as a categorical variable with four categories: high school‐level, vocational training, polytechnic degree, or university degree. Mothers also reported how many siblings the infant had. We primarily used sibling data from when the infant was 12 months old, and completed missing data with corresponding data from 3 months postpartum, and used number of siblings as a continuous variable in analyses.

Additionally, maternal psychological distress has been found to relate to both child communicative development and temperament, as well as mother's ratings of child behavior (Durbin and Wilson 2012; Hanington, Ramchandani, and Stein 2010; Reck et al. 2018; Sohr‐Preston and Scaramella 2006). To control for these potential effects in our sample, a maternal psychological distress composite score was computed by summing standardized sum scores from the Edinburgh Postnatal Depression Scale (EPDS, Cox, Holden, and Sagovsky 1987), completed at 12 months postpartum, and the anxiety subscale of the Symptom Checklist – 90 (SCL‐90, Derogatis, Lipman, and Covi 1973) at 6 months postpartum (correlation between the measures: *r* = 0.70). In one case where SCL‐90 data from 6 months were missing, we used the corresponding data from 3 months postpartum. The psychological distress score was used as a continuous covariate in analyses. To control for maternal language skills, we used the Wechsler Adult Intelligence Scale – Fourth Edition Verbal Comprehension subscale (WAIS‐IV VCI, Wechsler 2012).

### 2.4 Data Analysis Strategy

Data were analyzed using IBM SPSS versions 28–29 and Mplus 8.7 software with maximum likelihood estimation and robust standard errors (MLR) (Muthén and Muthén 1998–2017). SPSS was used for descriptive data analyses and zero‐order correlation analysis. In the study, *p*‐values below 0.05 were considered significant. Correction for multiple comparisons was done using the Benjamini–Hochberg false discovery rate procedure. To examine bivariate associations, Spearman's correlation coefficient was used since not all included variables were normally distributed. Data were missing in 11%–20% of cases for observed self‐regulation by gaze aversion, the 12‐month reported temperament factors, 14‐month communicative gesturing, and 30‐month vocabulary. For the remaining variables, data was missing in 0%–10% of the cases. According to Little's MCAR test, data were missing completely at random (chi‐square = 534.80, DF = 505, *p* = 0.17). Missing data were handled using full information maximum likelihood estimation (FIML) in Mplus.

Mplus was used to create and test path models involving emotional availability and temperament traits as predictors of communicative development. The MLR estimation method was used since not all variables were normally distributed. Model fit was assessed using the chi‐square test statistic, where a non‐significant test was regarded as an indicator of adequate model fit, along with Tucker‐Lewis index (TLI, cutoff > 0.95), comparative fit index (CFI, cutoff > 0.95), root mean square error of approximation (RMSEA, cutoff < 0.06), and standardized root mean square residual (SRMR, cutoff < 0.06) (Hu and Bentler 1999). Standardized coefficients (STDYX) were reported. First, a latent variable for emotional availability consisting of all the EA‐subscales (sensitivity, structuring, non‐intrusiveness, and non‐hostility) was created. Similarly, we created a latent variable for 14‐month communicative skills consisting of gesturing, expressive, and receptive vocabulary.

**FIGURE 1:**
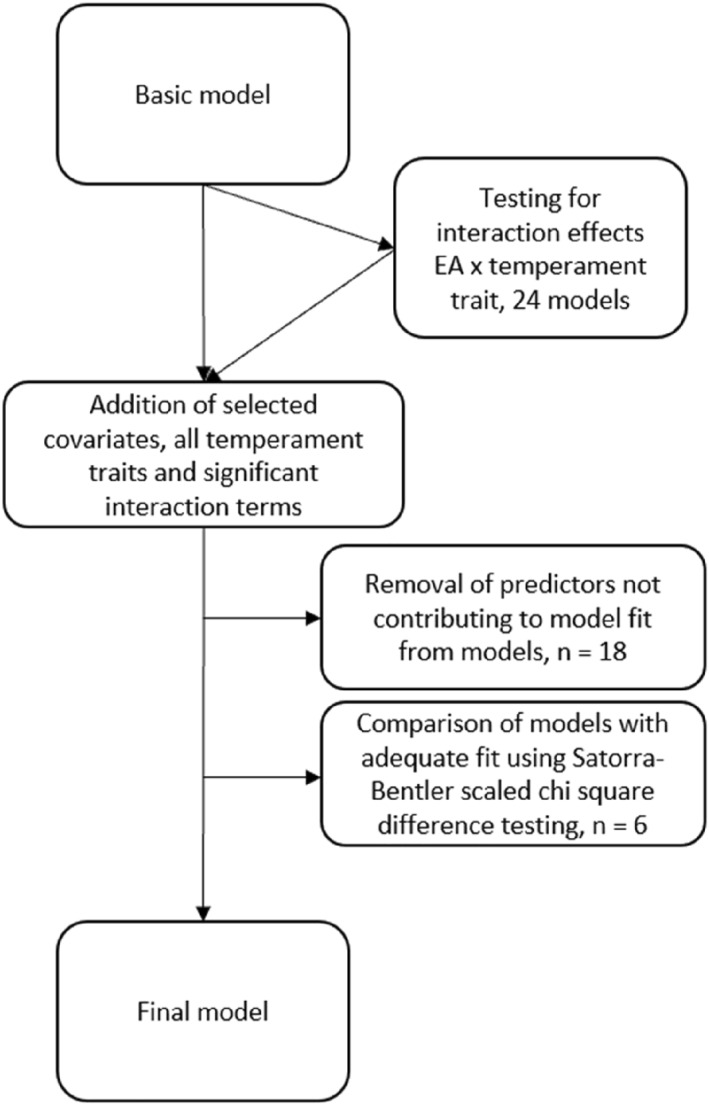
The model building process.

A flow chart depicting the model building process is provided in Figure 1. First, a basic model including paths between the emotional availability latent variable and the communication variables at 14 months and 30 months was created to investigate how emotional availability predicts communicative development. Second, interaction terms consisting of the emotional availability latent variable and each temperament trait respectively (observed variables for reported temperament traits at 6 and 12 months and 8‐month observational measures separately) were created using the XWITH‐command (Maslowsky, Jager, and Hemken 2015). Interaction terms were added to the basic model separately, accompanied by selected covariates, to test for moderation effects in analyses. Continuous covariates displaying significant zero‐order correlations with any of the 14‐month communicative skills (i.e., gestational age at birth), as well as child gender and maternal education level, were chosen as covariates in the models. Moderation was tested for 14‐month language and 30‐month vocabulary in separate models. In cases where the interaction term significantly predicted the 14‐month or 30‐month language variable in the basic model with the emotional availability latent and the temperament trait included as predictors, model fit was compared between the null model, without the interaction term, and the alternative model, including the interaction term, using a log‐likelihood ratio test with scaling correction factors (Satorra and Bentler 2010). The interaction effect was regarded as significant in cases where there was a significant improvement in the model fit in the models including the interaction term compared to the null model (i.e., the basic model with covariates but no interaction term), according to the log‐likelihood ratio test (Maslowsky, Jager, and Hemken 2015).

**FIGURE 2:**
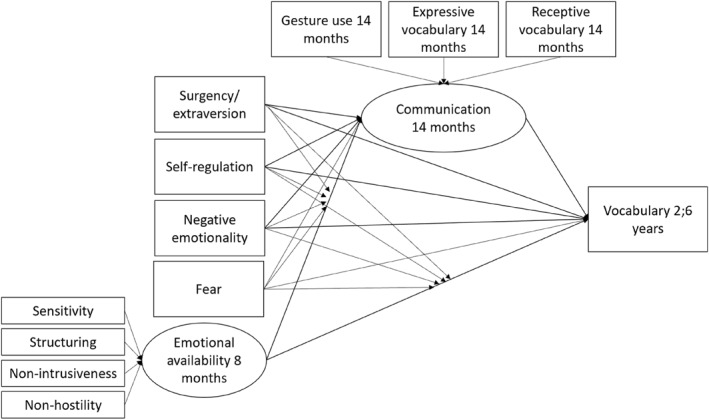
Hypothetical final model of associations between EA, temperament and communicative skills.

Third, a final model was built, initially including the emotional availability latent variable, all temperament traits (the observed reported temperament variables at 6 and 12 months, and observationally assessed observed variables at 8 months separately), the selected covariates mentioned above, as well as potential significant interaction terms as predictors of 14‐month and 30‐month language, and then improving the model by stepwise removing predictors not contributing to the model fit. When creating the basic and final models, possible competing models of adequate fit were to be compared using the Satorra–Bentler scaled chi‐square for chi‐square difference testing (Satorra and Bentler 2010). *N* = 151 for all models reported. The hypothetical final model is pictured in Figure 2.

## 3 Results

Descriptive statistics regarding the measures used in the study are displayed in Table 2.

**TABLE 2:** Descriptive statistics for the measures used in the study.

Measure (theoretical range)	*M* (*SD*)	Range
CDI		
Gesture use 14 months (100%)	54.6 (13.2)	9.1–87.9
Expressive vocabulary 14 months (100%)	2.8 (4.1)	0–35.5
Receptive vocabulary 14 months (100%)	36.7 (19.4)	1.8–91.3
Expressive vocabulary 30 months (100%)	71.1 (24.7)	2.0–100
EAS		
Sensitivity (1–7)	5.3 (1.3)	2–7
Structuring (1–7)	5.0 (1.5)	2–7
Non‐intrusiveness (1–7)	5.7 (1.4)	1–7
Non‐hostility (1–7)	6.1 (1.0)	3–7
Lab‐TAB		
Fear (0–14)	3.7 (2.3)	0–12.1
Attention (0–5)	3.7 (0.6)	0.8–4.7
Negative emotionality (0–11)	4.5 (2.0)	0.5–8.2
Self‐regulation by gaze aversion (0–60)	21.7 (10.3)	0–54
IBQ‐R 6 months (1–7)		
Surgency/extraversion	4.6 (0.8)	2.5–6.3
Negative emotionality	3.0 (0.8)	1.5–5.0
Emerging self‐regulation	5.3 (0.6)	3.4–6.6
Fear	2.6 (1.2)	1.0–6.0
IBQ‐R 12 months (1–7)		
Surgency/extraversion	5.1 (0.6)	3.6–6.3
Negative emotionality	3.3 (0.7)	1.6–4.8
Emerging self‐regulation	5.0 (0.7)	3.1–6.3
Fear	2.9 (1.2)	1.0–6.3

### 3.1 Zero‐Order Associations

Zero‐order correlations between study variables are reported in Table 3. Significant correlations were found between communicative skills and EA scales as well as some of the observed and reported temperament traits. Of the covariates, gestational age was positively correlated with all 14‐month communicative skills and was thus included as a covariate in model building.

**TABLE 3:** Zero‐order correlations between measures in the study.

	1.	2.	3.	4.	5.	6.	7.	8.	9.	10.	11.	12.	13.	14.	15.	16.	17.	18.	19.	20.	21.	22.	23.
1. Receptive vocabulary 14 months																							
2. Expressive vocabulary 14 months	0.39**																						
3. Gesture use 14 months	0.56**	0.32**																					
4. Vocabulary 30 months	0.43**	0.38**	0.45**																				
5. Negative emotionality 8 months	0.06	−0.06	−0.03	0.05																			
6. Fearfulness 8 months	0.12	0.12	0.18*	0.02	−0.00																		
7. Attention 8	−0.05	−0.07	−0.10	−0.08	0.09	−0.07																	
8. Regulation by gaze aversion 8 months	−0.21*	−0.24**	−0.17	−0.08	−0.07	−0.20*	−0.04																
9. Surgency/extraversion 6 months	0.31**	0.19*	0.37**	0.27**	0.02	−0.01	−0.06	−0.03															
10. Negative emotionality 6 months	0.01	−0.03	−0.01	0.04	0.17	0.11	−0.12	−0.04	0.24**														
11. Fear 6 months	0.09	0.14	0.12	0.08	0.04	0.07	−0.15	−0.03	0.18*	0.61**													
12. Emerging self‐regulation 6 months	0.12	0.11	0.21*	0.05	−0.18*	−0.14	0.00	−0.14	0.46**	−0.22*	0.01												
13. Surgency/extraversion 12 months	0.27**	0.16	0.46**	0.21*	0.09	−0.03	0.03	0.03	0.61**	−0.09	0.03	0.31**											
14. Negative emotionality 12 months	−0.06	−0.02	0.06	−0.01	0.18*	0.08	−0.24**	0.05	0.11	0.58**	0.34**	−0.13	0.15										
15. Fear 12 months	0.02	0.08	0.04	0.09	0.11	0.08	−0.17	0.20*	0.09	0.25**	0.41**	0.06	0.08	0.55**									
16. Emerging self‐regulation 12 months	0.21*	0.16	0.43**	0.17	−0.02	−0.05	0.02	−0.01	0.44**	−0.30**	−0.10	0.66**	0.39**	−0.15	0.13								
17. Maternal sensitivity 8 months	0.18*	0.05	0.11	0.10	−0.01	0.06	−0.03	−0.05	−0.20*	−0.16	−0.05	0.02	0.01	−0.17	−0.12	0.05							
18. Maternal structuring 8 months	0.19*	0.04	0.15	0.04	−0.00	−0.04	−0.02	−0.06	−0.14	−0.21*	0.01	0.11	0.03	−0.08	−0.05	0.11	0.71**						
19. Maternal non‐intrusiveness 8 months	0.27**	0.18*	0.14	0.18*	−0.05	0.03	−0.04	−0.05	0.00	0.04	0.13	−0.02	0.13	0.07	0.05	0.10	0.53**	0.38**					
20. Maternal non‐hostility 8 months	0.20*	−0.01	0.04	0.01	−0.02	0.06	0.01	−0.08	−0.10	−0.01	0.05	−0.02	−0.01	0.00	0.02	0.10	0.59**	0.39**	0.49**				
21. Number of siblings	−0.11	−0.04	0.05	−0.21**	0.06	0.06	−0.03	−0.10	−0.08	0.00	0.12	0.00	−0.03	0.16**	0.07	−0.01	0.13	0.09	0.16	0.18*			
22. Gestational age	0.20**	0.15*	0.14*	0.11	0.03	0.02	0.08	−0.11	0.10	0.08	0.00	0.04	0.02	−0.00	0.03	0.09	−0.04	−0.05	0.08	0.13	−0.06		
23. Psychological distress	−0.09	−0.02	−0.08	−0.13*	0.05	0.03	−0.02	−0.05	−0.02	0.29**	0.13*	−0.08	−0.00	0.32**	0.06	−0.14*	−0.09	−0.07	−0.01	−0.14	−0.07	0.09	
24. Maternal verbal intelligence	−0.04	0.15	0.07	0.27*	−0.03	0.02	−0.01	−0.09	−0.21	−0.05	0.10	−0.11	−0.13	0.08	0.14	0.03	−0.05	0.01	0.18	−0.08	0.24*	−0.01	−0.18

*p < 0.05, **p < 0.01.

### 3.2 SEM‐Results

#### 3.2.1 Path Model of the Association Between Emotional Availability and Early Communicative Development

**FIGURE 3:**
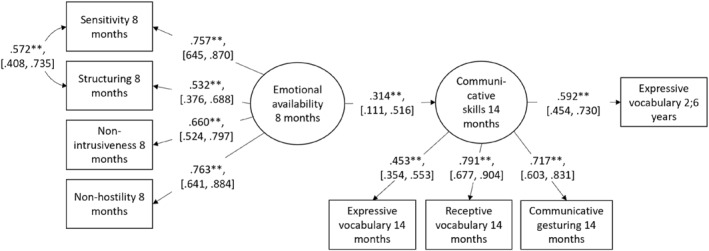
Basic path model describing associations between emotional availability and communicative development. *N* = 151. *χ* ^2^(18) = 13.6*, p* = 0.757, RMSEA = 0.00, CFI = 1.00, TLI = 1.00, SRMR = 0.03. Standardized coefficients. 95% CI for estimates are reported in brackets. **p < 0.01.

First, measurement models were tested for two latent constructs: communicative development and emotional availability. The model fit for communicative development was *χ* ^2^(2) = 0.58, *p* = 0.748, RMSEA = 0.00, CFI = 1.00, TLI = 1.00, SRMR = 0.02. The best‐fitting measurement model for emotional availability was one where all emotional availability scales (sensitivity, structuring, non‐intrusiveness, and non‐hostility) were used as a single latent variable (*χ* ^2^(1) = 0.08, *p* = 0.781, RMSEA = 0.00, CFI = 1.00, TLI = 1.00, SRMR = 0.00). Sensitivity and structuring were allowed to correlate to improve model fit. We also tried creating different latent variables for positive (sensitivity and structuring) and negative parenting (non‐intrusiveness and non‐hostility); but the high correlation between these two latent variables would have resulted in high risk of multicollinearity. We found no significant direct prediction of emotional availability on expressive language at 30 months, resulting in leaving out that path from the final model. Rather, the emotional availability latent variable was directly associated with the 14‐month communicative skills latent variable, which in turn was significantly associated with 30‐month vocabulary (*χ* ^2^(18) = 13.6*, p* = 0.757, RMSEA = 0.00, CFI = 1.00, TLI = 1.00, SRMR = 0.03). The indirect effect between emotional availability and 30‐month vocabulary through 14‐month communication was significant (*b* = 0.19, *p* = 0.003). The basic model is depicted in Figure 3.

#### 3.2.2 Moderation Effects of Temperament Traits on the Association Between Emotional Availability and Communicative Development

In the test of moderation by temperament on associations between EA and communicative development, individual models were built for all temperament traits respectively. That is, interaction terms were created for all individual temperament traits at 6, 8, and 12 months and tested in separate models along with the EA latent and the corresponding temperament main effect. There was some evidence of an interaction effect of negative emotionality by emotional availability. More specifically, the interaction term of reported negative emotionality at 6 months and emotional availability was associated with 14‐month communicative skills when added to a model consisting of the basic model (Figure 3) along with the main effects in the interaction term and the chosen covariates (fit indices for the null model without the interaction term: *χ* ^2^(40) = 56.4*, p* = 0.04, RMSEA = 0.05, CFI = 0.95, TLI = 0.94, SRMR = 0.06, *N* = 151). The Satorra–Bentler chi‐square test comparing the null model and the model including the interaction term (Δ *χ* ^2^(−1) = 6.2*, p* = 0.01) indicated that the fit of the model including the interaction term was superior to that of the null model. Additionally, the interaction term consisting of reported negative emotionality at 12 months of age and the emotional availability latent significantly predicted 30‐month vocabulary in a corresponding model (null model: *χ* ^2^(40) = 66.9*, p* = 0.005, RMSEA = 0.07, CFI = 0.92, TLI = 0.90, SRMR = 0.09, *N* = 151). Similarly, the Satorra–Bentler chi square test comparing the null model and model including the interaction term (Δ *χ* ^*2*^(−1) = 4.9, *p* = 0.03) indicated that the fit of the model including the interaction term exceeded that of the null model. The interactions suggested that low negative emotionality at 6 months combined with high emotional availability was related to relatively better communicative skills at 14 months, whereas high negative emotionality at 12 months combined with high emotional availability predicted better vocabulary at 30 months. However, the significant interactions did not survive the correction for multiple comparisons (diagrams shown in Supporting Informations S1 and S2). No additional interaction terms were significant.

#### 3.2.3 Final Path Model of Relations Between Emotional Availability, Temperament Traits, Covariates and Early Communicative Development

**FIGURE 4:**
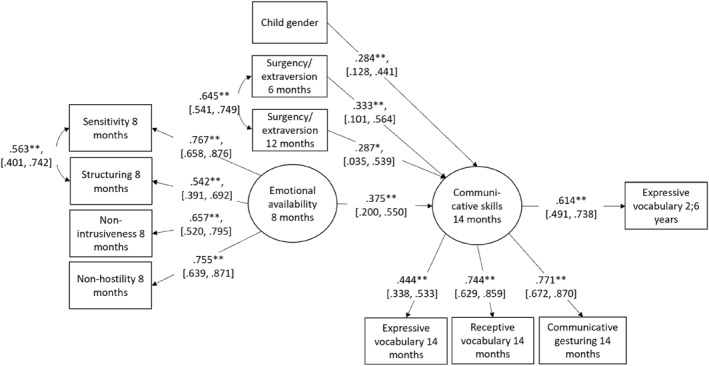
Final path model describing associations between emotional availability, infant temperament, covariates and communicative development. *N* = 151. Standardized coefficients. 95% CI for estimates are reported in brackets. *p < 0.05, **p < 0.01.

First, all temperament traits and selected covariates were added to the model. Paths to both 14‐month communicative skills and 30‐month expressive vocabulary were tested for all temperament traits. Non‐significant predictors were eliminated from the model one by one until models with acceptable model fit were achieved. The satisfactory models were compared using the Satorra–Bentler chi‐square difference test (Satorra and Bentler 2010) to choose the best model. The final model (*χ* ^2^(39) = 46.3*, p* = 0.1978, RMSEA = 0.04, CFI = 0.98, TLI = 0.98, SRMR = 0.05) is presented in Figure 4. Communicative skills at 14 months were positively predicted by higher emotional availability and reported surgency/extraversion at both 6 and 12 months of age, as well as by female gender. The only significant path to 30‐month expressive vocabulary was from 14‐month communicative skills.

## 4 Discussion

The aim of the current study was to examine the relations between caregiving quality, infant temperament attributes, and communicative development, and to explore possible moderation effects between temperament and emotional availability in explaining communicative development. We found evidence that maternal EA in infancy is associated with favorable early toddlerhood communicative development, which in turn is positively related to 30‐month vocabulary. Furthermore, higher infant surgency/extraversion positively contributed to communicative development, but no convincing evidence was found for temperament being a moderator between caregiving quality and language outcomes. Female infants also tended to have stronger early communicative skills, corresponding to the tendency for advantages of female sex in early language development frequently reported and discussed in the literature (e.g., Lange and Zaretsky 2021; Rinaldi et al. 2023). To our knowledge, this study is among the first to examine the relations between caregiving quality in the comprehensive EA framework and child communicative development, accompanied by child temperament as a moderating factor.

### 4.1 Caregiving Quality and Communicative Development

In accordance with our hypothesis, the latent variable of maternal EA comprising different aspects of sensitive caregiving in infancy positively predicted child communicative skills at 14 months of age. In other words, high‐quality caregiving in infancy predicted better communicative skills in early toddlerhood. This finding concurs with the current literature (Leigh, Nievar, and Nathans 2011; Nozadi et al. 2013; Tamis‐LeMonda, Bornstein, and Baumwell 2001). However, the current study offers unique new contributions to existing knowledge by showing that relations are found also when considering emotional availability and communicative skills as latent variables.

The emotional availability latent variable used involves all EA scales (sensitivity, structuring, non‐intrusiveness, and non‐hostility), and thereby comprises a more overarching concept of caregiving quality, whereas previous studies have involved aspects of caregiving independently and have mainly studied sensitivity/responsiveness (Leigh, Nievar, and Nathans 2011; Nozadi et al. 2013; Tamis‐LeMonda, Bornstein, and Baumwell 2001), and in some cases intrusiveness (Bruce et al. 2022; Cabrera, Shannon, and Tamis‐LeMonda 2007; Keown, Woodward, and Field 2001; Pungello et al. 2009; Tamis‐LeMonda et al. 2004). It seems theoretically plausible that including structuring and non‐hostility in the concept of caregiving quality further enhances the relationship with communicative development due to the nature of these subscales. Structuring is the parent's ability to support the child to learn and be active by adapting the situation according to the child's abilities, allowing autonomy but offering support when needed (Saunders et al. 2015). Possibly, this also leads to using age‐appropriate and contextually relevant child‐directed language. Furthermore, non‐hostility refers to the parent's regulation of their own negative emotions and behaviors, for example boredom, frustration or aggression, towards the child (Saunders et al. 2015), which is likely to also be accompanied by more positive and engaged communication from the parent and encourage communicative initiatives from the child. In summary, in relation to aspects of caregiving previously studied, EA has more emphasis on emotional aspects (Biringen 2000) rather than a communicative focus. For example, EA does not involve a direct measure of responsiveness to or development of infant communicative initiatives. Taken together, high overall EA is indicative of a warm, sound, invested, mutually enjoyable relationship between parent and child (Biringen and Easterbrooks 2012). Consequently, the parent can be highly sensitive with the infant using mostly eye contact, gestures, facial expressions, and tone of voice, without necessarily offering rich verbal stimulation. It is, however, noteworthy that the current design with a latent variable does not allow drawing conclusions regarding which fine‐grained aspects of EA are particularly related to communicative development. Hence, it may be either the emotional or interactional aspects or both intertwined that underlie the association with communicative skills. This could be examined further in future studies.

Regarding communication as an outcome, it has most often been assessed as either only expressive (Leigh, Nievar, and Nathans 2011; Tamis‐LeMonda, Bornstein, and Baumwell 2001) or both expressive and receptive language skills (Keown, Woodward, and Field 2001; Pungello et al. 2009). In rare cases, preverbal communication such as gesturing (Bruce et al. 2022) or other communicative dimensions such as symbolic play (Bornstein and Tamis‐Lemonda 1997) have been considered. In the current study, we aimed to have a more general perspective on early communicative skills by using a latent variable involving gesturing, expressive, and receptive vocabulary, which all loaded onto the same latent factor. Including gesturing in the assessment of this age group is important, as nonverbal communication constitutes a large part of communication at this age and is tightly related to later verbal communication (Iverson and Goldin‐Meadow 2005). Consequently, the current findings suggest that relations do not exist only between specific subscales of caregiving quality and dimensions of communication, but also when considering the more general phenomena of emotional availability and communicative skills.

There was no significant direct longitudinal prediction of emotional availability on 30‐month vocabulary. However, there was a positive indirect longitudinal association between EA and 30‐month vocabulary through communicative skills at 14 months. In a previous longitudinal study with consecutive assessments of both maternal sensitivity and child language from infancy up to 3;0 years, Leigh, Nievar, and Nathans (2011) found both significant longitudinal and concurrent associations between sensitivity and language, as well as significant associations between earlier sensitivity and language on the corresponding dimensions later on in the same statistical model. This led them to conclude that the longitudinal associations between expressive language and maternal sensitivity are relatively stable over time. The strong associations between maternal sensitivity and language in toddlerhood, along with the strong prediction of early sensitivity on later sensitivity, highlight the importance of sensitivity starting from the earliest days (Leigh, Nievar, and Nathans 2011). These findings, together with our study, indicate that early caregiving quality can be of particular importance for stimulating a favorable start for the communicative development. Later in childhood, favorable language development may be more strongly related to the child's previous language skills and more direct verbal language stimulation, resulting in diminishing direct effects of early caregiving quality on later language skills.

### 4.2 Caregiving Quality, Temperament, and Communicative Development

In the final model, there were direct positive effects of emotional availability, surgency/extraversion, and female gender on 14‐month communicative development. The main effect of surgency/extraversion predicting communication corresponds well with previous research, where temperament traits related to extraversion and positive emotionality are frequently reported to have positive associations with language (Laake and Bridgett 2014) and also gesturing in our previous study (Ollas et al. 2020). Suggested explanations are that the sociability and approach tendencies related to infant surgency/extraversion elicit more communication experiences, and the trait perhaps encourages caregivers to interact with the infant, resulting in more language and communication stimulation (Laake and Bridgett 2014). Contrary to the hypothesis, no significant moderation effects of any temperament trait on associations between caregiving quality and communicative development were found after taking into account multiple comparisons. Thus, our findings do not support the hypothesis that the relation between caregiving quality, here measured as EA, and communicative development is moderated by infant temperament. They rather suggest that infant surgency/extraversion has an independent positive influence on infant communicative development, and that high emotional availability is positive for communicative development even when controlling for infant temperament.

Previous studies on interaction effects have differed in their methodology and are not directly comparable, but seem to suggest that higher positive and/or negative emotionality is beneficial for language development when paired with high responsiveness (Karrass and Braungart‐Rieker 2003; Laake and Bridgett 2018; Nozadi et al. 2013), whereas parental intrusiveness paired with high emotionality has negative associations with language, but can have positive effects if paired with low negative emotionality (Laake and Bridgett 2018). The studies differed regarding which aspects of caregiving and language were studied, and some of the associations were found for boys only. Considering this, it is not surprising that no significant effects were found in our study with more general perspectives on both caregiving and communication. Additionally, our study was conducted in a relatively low risk sample with relatively high overall EA. This results in lower power for detecting effects of the more extreme variations within caregiving. Possibly, caregiving quality and temperament would be more closely related in samples with higher risks in terms of caregiving, SES, psychosocial welfare, or child communicative development challenges. For example, it seems plausible caregivers low in EA would be more likely to ignore or communicate in a negative tone with infants high in negative emotionality. Taken together, the exploration of interaction effects between temperament and caregiving quality as predictors of communicative development is still at an early stage, and more studies in the field are needed.

As discussed previously, EA as a concept does not involve specific assessment of maternal communicative responses, but rather has an emotional focus (Biringen 2000). Thereby, high EA is not automatically accompanied by high verbal or communicative stimulation, but may serve as an enforcement of communicative skills through creating an enjoyable relationship and positive experiences of interaction. Instead, perhaps surgency/extraversion in the infant specifically encourages more verbal interaction and lexical variability outside of the context of basic caregiving quality. For example, a study focusing on maternal lexical input, rather than overall caregiving quality, found lexical input to moderate associations between infant orienting and toddlerhood language (Spinelli et al. 2018). Possibly, the verbal dimensions of parent‐child interaction could be more interrelated with other temperamental dimensions as well, such as surgency/extraversion in this study. Additionally, surgency/extraversion may be efficient in supporting communicative development and language learning in other contexts, outside of interaction with the primary caregiver. Surgency/extraversion may have a larger influence on eliciting communication with persons besides the primary caregivers. For example, other persons may be more inclined to interact with infants that spontaneously show more joy and interest in interacting, providing them with more diverse communicative experiences. Indeed, Sheinkopf et al. (2017) found that infant positive affect towards the examiner, but not towards the mother, in 4‐month‐old infants predicted their verbal IQ at 4;6 years of age.

In sum, results of the moderation analysis suggest that emotional availability has an independent effect on communicative development and further highlight the importance of emotional aspects of caregiving in supporting communicative development in infants across variable temperament profiles. However, future studies need to continue attempts to replicate our findings. Extending such work using samples from more diverse backgrounds, higher number of participants with established parenting risks (high socioeconomic or psychosocial risk), or risks for atypical communication development will be particularly important.

### 4.3 Strengths, Limitations and Directions for Future Research

The current study has several notable strengths. It addresses several gaps in the existing literature on associations between caregiving quality, infant temperament, and communicative development. Furthermore, the sample is relatively large and is part of a prospective birth cohort study that has a sample relatively representative of the Finnish population. We also employed both objective and parent reported measures of child temperament in accordance with recommendations for studying temperament (Stifter, Willoughby, and Towe‐Goodman 2008) and we utilized SEM to create latent factors allowing the study of more general phenomena of emotional availability and communicative development, and offering a longitudinal perspective into toddlerhood vocabulary. However, there are several limitations. The use of latent variables of EA and communicative skills can be considered strengths of the study design. However, the use of a latent variable does not allow for assessing effects of more fine‐grained aspects of EA and communicative skills. Particularly in the case of EA, future studies should assess how the different EA scales individually relate to communicative development, as this is currently not known. Moreover, although our sample size is similar to other studies in the field, there may be a risk for false negatives regarding smaller effects. Longitudinal studies involving assessments of EA later in development and more comprehensive assessments of communicative development similar to the study by Leigh, Nievar, and Nathans (2011) are needed. Also, direct assessments of maternal language input would be important to include in future studies to distinguish between the influence of maternal emotional availability and linguistic input in predicting child communicative development. Additionally, the relatively low risk level for both EA and child communicative development in the sample indicates that caution is needed in generalizing the findings reported in this study to higher risk populations. Considering the assessment of child EA in addition to maternal EA would have been valuable and will be considered in future studies. In the current study, only maternal caregiving quality was studied. This can be considered a limitation since fathers and other frequent communication partners in the child's life have been found to have associations with child language outcomes as well (Cabrera, Shannon, and Tamis‐LeMonda 2007; Rost and McMurray 2009; Seidl, Onishi, and Cristia 2014). Additionally, assessments of both home languages were not available for the bilingual children, resulting in a potentially unrepresentative assessment of their communicative ability. However, the number of bilingual children was relatively low, 8 out of the total sample of 151. Furthermore, communicative development was only assessed by parent report. While this is a common and valid method in studies on toddler language skills (Feldman et al. 2000; Stolt et al. 2009), future studies should complement parental report with direct observation of child communicative behaviors. Finally, we did not involve an observational assessment of child surgency/extraversion, and future studies should consider doing so.

## 5 Conclusions

The current study provides further support for caregiving quality playing an important role in scaffolding communicative development in infancy and early toddlerhood, and adds to the knowledge by observing this association in a context of studying caregiving as a general measure of emotional availability and communicative development, combining several important infant milestones: gesturing, expressive, and receptive vocabulary. Additionally, maternal emotional availability and infant surgency/extraversion had independent associations with communicative outcomes. No compelling evidence of infant temperament moderating the association between emotional availability and communicative development was identified in the current study. Consequently, the results highlight the importance of supporting emotional availability starting from early infancy in all caregiver‐child dyads to foster young children's communicative development.

## Author Contributions

**Denise Ollas‐Skogster:** conceptualization, data curation, formal analysis, funding acquisition, methodology, writing–original draft. **Riikka Korja:** investigation, writing–review & editing. **Akie Yada:** data curation, formal analysis. **Elina Mainela‐Arnold:** conceptualization, writing–review & editing. **Hasse Karlsson:** funding acquisition, project administration, resources. **David J. Bridgett:** conceptualization, writing–review & editing. **Pirkko Rautakoski:** conceptualization, funding acquisition, supervision, writing–review & editing. **Linnea Karlsson:** conceptualization, funding acquisition, supervision, writing–review & editing. **Saara Nolvi:** conceptualization, formal analysis, funding acquisition, methodology, supervision, writing–original draft.

## Conflicts of Interest

The authors declare no conflicts of interest.

## Supporting information

Supporting Information S1

Supporting Information S2

## Data Availability

Access to the FinnBrain cohort data can be acquired by contacting PI Prof Linnea Karlsson (linnea.karlsson@utu.fi) to establish research collaboration. Sharing data outside research collaboration is precluded by Finnish national legislation on personal data protection and the ethics regulations of the FinnBrain cohort.
