# Supplementary material for: Maternal Emotional Availability Supports Child Communicative Development Regardless of Child Temperament—Findings From the FinnBrain Birth Cohort Study
Source: Infancy. 2025 Jan 24;30(1):e12649. doi: 10.1111/infa.12649 (PMC11758768; doi:10.1111/infa.12649)

Supplement 1

*Model of the interaction term between 6-month negative emotionality and emotional availability predicting 14-month communicative skills*


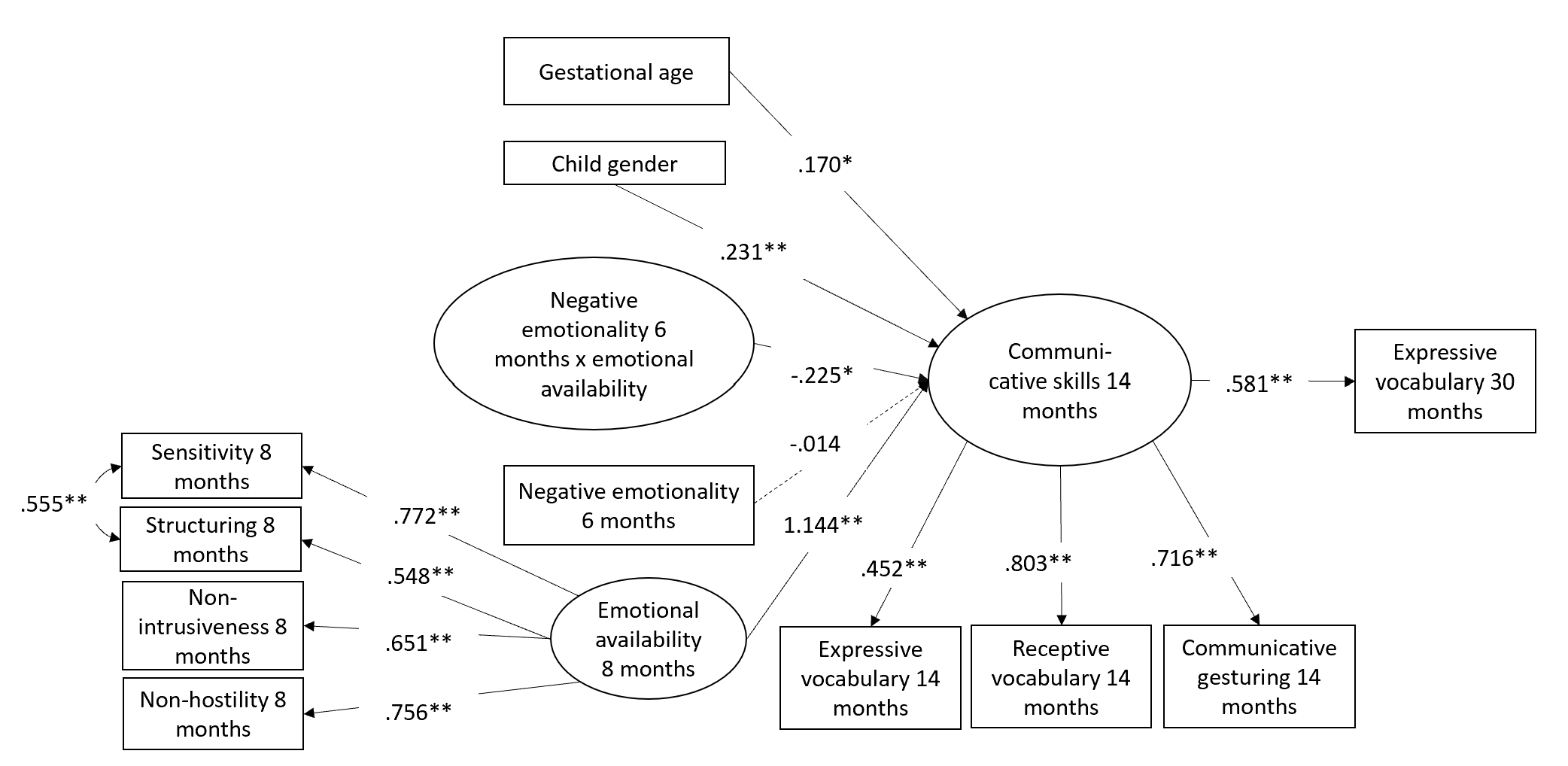

Supplement: Supplementary file 1 — Supporting Information S1 [file INFA-30-0-s002.docx]
