# Supplementary material for: Maternal Emotional Availability Supports Child Communicative Development Regardless of Child Temperament—Findings From the FinnBrain Birth Cohort Study
Source: Infancy. 2025 Jan 24;30(1):e12649. doi: 10.1111/infa.12649 (PMC11758768; doi:10.1111/infa.12649)

Supplement 2

*Model of the interaction term between 12-month negative emotionality and emotional availability predicting 30-month expressive vocabulary*


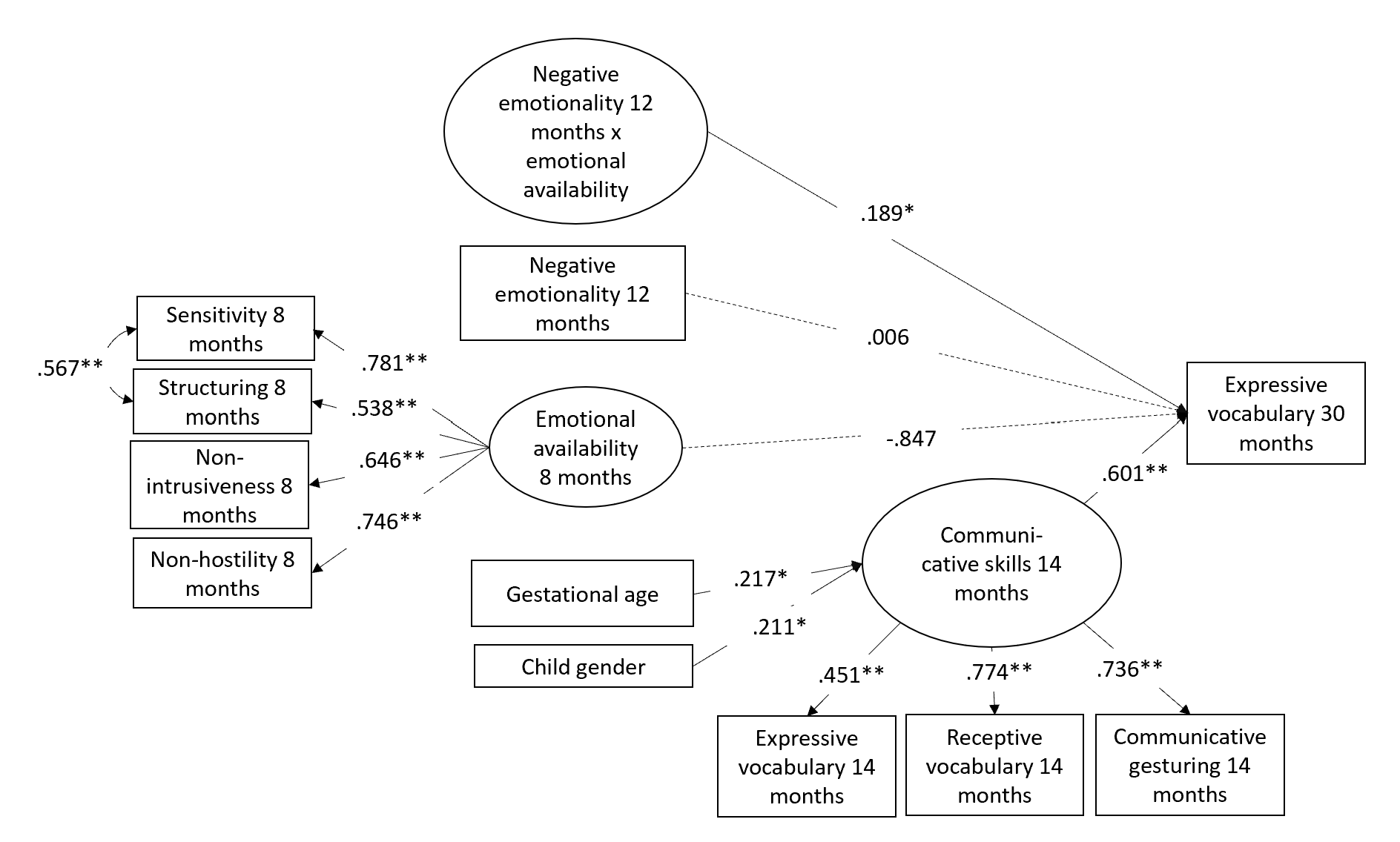

Supplement: Supplementary file 2 — Supporting Information S2 [file INFA-30-0-s001.docx]
